# Machine learning based risk prediction for Parkinson's disease with nationwide health screening data

**DOI:** 10.1038/s41598-022-24105-9

**Published:** 2022-11-14

**Authors:** You Hyun Park, Jee Hyun Suh, Yong Wook Kim, Dae Ryong Kang, Jaeyong Shin, Seung Nam Yang, Seo Yeon Yoon

**Affiliations:** 1 Department of Biostatistics, Yonsei University, Seoul, Korea; 2 Department of Rehabilitation Medicine, College of Medicine, Ewha Womans University, Seoul, Korea; 3 Department and Research Institute of Rehabilitation Medicine, Yonsei University College of Medicine, Seoul, Republic of Korea; 4 Department of Precision Medicine & Biostatistics, Yonsei University Wonju College of Medicine, Wonju, Korea; 5 Department of Preventive Medicine and Public Health, Yonsei University College of Medicine, Seoul, Republic of Korea; 6 Department of Physical Medicine and Rehabilitation, Korea University Guro Hospital 148, Gurodong-Ro, Guro-Gu, Seoul, 08308 Republic of Korea

**Keywords:** Neurology, Risk factors

## Abstract

Although many studies have been conducted on machine learning (ML) models for Parkinson’s disease (PD) prediction using neuroimaging and movement analyses, studies with large population-based datasets are limited. We aimed to propose PD prediction models using ML algorithms based on the National Health Insurance Service-Health Screening datasets. We selected individuals who participated in national health-screening programs > 5 times between 2002 and 2015. PD was defined based on the ICD-code (G20), and a matched cohort of individuals without PD was selected using a 1:1 random sampling method. Various ML algorithms were applied for PD prediction, and the performance of the prediction models was compared. Neural networks, gradient boosting machines, and random forest algorithms exhibited the best average prediction accuracy (average area under the receiver operating characteristic curve (AUC): 0.779, 0.766, and 0.731, respectively) among the algorithms validated in this study. The overall model performance metrics were higher in men than in women (AUC: 0.742 and 0.729, respectively). The most important factor for predicting PD occurrence was body mass index, followed by total cholesterol, glucose, hemoglobin, and blood pressure levels. Smoking and alcohol consumption (in men) and socioeconomic status, physical activity, and diabetes mellitus (in women) were highly correlated with the occurrence of PD. The proposed health-screening dataset-based PD prediction model using ML algorithms is readily applicable, produces validated results, and could be a useful option for PD prediction models.

## Introduction

Parkinson’s disease (PD) is a progressive neurological disorder associated with progressive neuronal loss of the substantia nigra and other brain structures and is characterized by tremor, bradykinesia, rigidity, and postural instability^1^. PD is an age-related and the second most common neurodegenerative condition. The prevalence of PD increases in the aging population, thus increasing the economic burden on the society^2–4^. The cardinal motor symptoms of PD are identified relatively late in the pathological process (i.e., when approximately 50% of dopaminergic neurons are lost in the substantia nigra); thus, PD diagnosis is often delayed^5,6^. Early detection or prediction of PD could make early pharmacological and non-pharmacological management possible, which could slow its progression. The benefits of early prediction and management of PD would affect not only the individual (and their families) but also the wider society and research community.

The diagnosis of PD is commonly based on medical observations and the assessment of clinical signs, such as resting tremor, bradykinesia, rigidity, and postural instability^7^. Recently, machine learning (ML) techniques have been increasingly applied in the healthcare sector, including the detection of PD^8^. For the early detection of PD, ML models have been applied to multiple data modalities, including movement, neuroimaging, and voice and handwriting patterns^9^. However, ML studies on PD prediction based on a large population-based dataset are scarce^10^. To our knowledge, there has been only one study on ML-based PD prediction using administrative claims data, and it contained 89,790 patients with PD^10^. In Korea, all insured adults aged ≥ 40 years are eligible for a general health-screening program that is biennially conducted, and the results are stored in the National Health Insurance Service-Health Screening (NHIS-healS) database^11^. Applying ML algorithms for PD prediction using the NHIS-healS database could be a cost-effective method because it uses existing data, thus negating the effort of new data collection. The NHIS-healS database has a large number of participants and includes various factors that could be related to the occurrence of PD, including demographic and anthropometric factors, socioeconomic status (SES), and comorbidities.

Lifestyle habits modulate the risk of PD^12^; however, since lifestyle factors change over time, it would be difficult to predict the occurrence of PD using baseline values of lifestyle factors. Therefore, in this study, we used health-screening data (measured more than five times) and attempted to elucidate how time-varying variables, such as lifestyle factors, anthropometric factors, and laboratory data, influence PD occurrence using ML algorithms. We aimed to construct data-driven ML models for PD prediction using repeatedly measured health-screening data and identified variables associated with PD occurrence stratified by sex. We also attempted to determine how the predictive performance of ML algorithms changed according to various factors, including demographic and anthropometric factors, SES, and comorbidities.

## Results

### Participant characteristics

Table 1 presents the demographic and medical characteristics of the PD group at the time of diagnosis and those of the comparison group at the final follow-up. The follow-up duration of the PD group was 3783.32 ± 615.29 days and that of the comparison group was 4602.66 ± 356.57 days. There were no significant differences in age between the two groups, indicating that age-matching was performed appropriately. There were no significant differences in sex between the two groups.

**Table 1:** Characteristics of study participants.

	Parkinson’s disease group		Comparison group		*P value*
	(n = 1102)		(n = 1102)		
	n	%	n	%	
**Age**					
Mean (SD)	70.91 (8.41)		70.91 (8.41)		1.000
40–49	7	0.64	7	0.64	1.000
50–59	128	11.62	128	11.62	
60–69	273	24.77	273	24.77	
70–79	531	48.19	531	48.19	
80 ≥	163	14.79	163	14.79	
**Sex**					
Male	597	54.17	610	55.35	0.5780
Female	505	45.83	492	44.65	
**Residential area**					
Urban	426	38.66	413	37.48	0.5685
Rural	676	61.34	689	62.52	
**Insurance type**					
NHI, self-employees	257	23.32	294	26.68	0.1788
NHI, employees	825	74.86	791	71.78	
Medical aid	20	1.81	17	1.54	
**Income level**					
Lowest	156	14.16	174	15.79	0.2091
Low-middle	206	18.69	204	18.51	
Middle-high	282	25.59	310	28.13	
Highest	458	41.56	414	37.57	
**Body mass index**					
Mean (SD)	23.69 (3.14)		24.09 (3.18)		0.0025
< 18.5	17	1.54	10	0.91	0.0651
18.5–23	395	35.84	340	30.85	
23–25	317	28.77	337	30.58	
25–30	349	31.67	378	34.30	
≥ 30	24	2.18	37	3.36	
**Systolic blood pressure**					
Mean (SD)	127.7 (15.87)		128.1 (12.24)		0.5756
**Diastolic blood pressure**					
Mean (SD)	76.71 (10.00)		76.85 (9.99)		0.7284
**Fasting glucose**					
Mean (SD)	104.6 (25.68)		103.8 (24.01)		0.4872
**Total cholesterol**					
Mean (SD)	186.0 (39.30)		190.9 (38.13)		0.0035
**Hemoglobin**					
Mean (SD)	13.43 (1.55)		13.65 (1.56)		0.0011
**Smoking**					
No	796	72.43	738	67.21	0.0178
Ex-smoker	212	19.29	239	21.77	
Current smoker	91	8.28	121	11.02	
**Alcohol consumption (per 1 week)**					
No	380	34.89	230	21.26	< 0.0001
≤ 3	685	62.90	852	78.74	
≥ 4	24	2.20	68		
**Physical activity (per 1 week)**					
≤ 4	730	66.24	665	60.34	0.0041
≥ 5	372	33.76	437	39.66	
**Hypertension**	781	70.87	744	67.51	0.0878
**Dyslipidemia**	578	52.45	588	53.36	0.6696
**Diabetes mellitus**	521	47.28	442	40.11	0.0007
**Ischemic heart disease**	337	30.58	328	29.76	0.6762
**Osteoporosis**	460	41.74	432	39.20	0.2243
**Congestive heart failure**	172	15.61	188	17.06	0.3566
**Peripheral vascular disease**	163	14.79	137	12.43	0.1063
**Cerebrovascular disease**	582	52.81	377	34.21	< .0001
**Dementia**	253	22.96	133	12.07	< 0.0001
**Chronic pulmonary disease**	741	67.24	791	71.78	0.0207
**Rheumatologic disease**	198	17.97	200	18.15	0.9118
**Peptic ulcer disease**	776	70.42	764	69.33	0.5775
**Mild liver disease**	459	41.65	499	45.28	0.0857
**Hemiplegia or paraplegia**	58	5.26	35	3.18	0.0148
**Renal disease**	30	2.72	45	4.08	0.0780
**Moderate or severe liver disease**	14	1.27	16	1.45	0.7131
**Metastatic solid tumor**	15	1.36	31	2.81	0.0171
**Ankylosing spondylitis**	25	2.27	26	2.36	0.8873
**Gout**	56	5.08	80	7.26	0.6989
**Irritable bowel syndrome**	629	57.08	620	56.26	0.0233
**Inflammatory bowel disease**	19	1.72	19	1.72	1.0000
**Constipation**	463	42.01	315	28.58	< 0.0001

Patients with PD had a lower proportion of current smokers and were less likely to drink alcohol or perform regular physical activity (PA); had lower mean body mass index (BMI), total cholesterol, and hemoglobin (Hb) levels; and had a higher prevalence (P < 0.05) of diabetes mellitus, cerebrovascular disease, dementia, irritable bowel syndrome, and constipation and lower prevalence (P > 0.05) of chronic pulmonary disease and metastatic solid tumors than those without PD.

### Predictive model for PD development

For predictive models, sex, SES (residential area, insurance type, and income level), anthropometric data (BMI, systolic and diastolic blood pressure), laboratory data (fasting glucose, total cholesterol, and Hb), lifestyle factors (smoking, alcohol consumption, and PA), and 22 comorbidities (such as hypertension, dyslipidemia, and diabetes mellitus) were evaluated (Table 1). For ML algorithms, we developed the following three predictive models based on different risk factors for PD development: model 1 included 26 variables, such as sex, SES, and comorbidities; model 2 included 29 variables, such as sex, SES, comorbidities, and lifestyle factors; and model 3 included 35 variables, such as sex, SES, comorbidities, lifestyle factors, and anthropometric and laboratory data. The area under the receiver operating characteristic curve (AUC) for each model ranged from 0.661 to 0.779 (Table 2). The model performance generally increased as more variables were included in the ML algorithms, and most ML algorithms demonstrated the best performance in model 3.

**Table 2:** Comparison of area under the receiver operating characteristic curve (AUC) by Machine Learning Algorithms in Parkinson’s disease prediction according to different included variables.

	Logistic regression	Random forest	Neural network	GBM	Decision tree	Naïve Bayes	XGBoost
Model 1	0.696 (0.646–0.746)	0.674 (0.614–0.734)	0.713 (0.660–0.766)	0.691 (0.642–0.741)	0.704 (0.651–0.757)	0.661 (0.624–0.706)	0.682 (0.629–0.724)
Model 2	0.702 (0.644–0.749)	0.691 (0.633–0.749)	0.767 (0.724–0.814)	0.764 (0.708–0.818)	0.713 (0.661–0.764)	0.674 (0.631–0.717)	0.710 (0.658–0.759)
Model 3	0.728 (0.676–0.777)	0.731 (0.679–0.783)	0.779 (0.732–0.826)	0.766 (0.717–0.815)	0.709 (0.668–0.759)	0.687 (0.649–0.725)	0.722 (0.679–0.765)

Model 1: Including sex, SES and comorbidities.

Model 2: Including sex, SES, comorbidities and lifestyle factors.

Model 3: Including sex, SES, comorbidities, lifestyle factors, and anthropometric and laboratory data.

*GBM* gradient boosting machines, *XGBoost* eXtreme gradient boosting.

**Figure 1:**
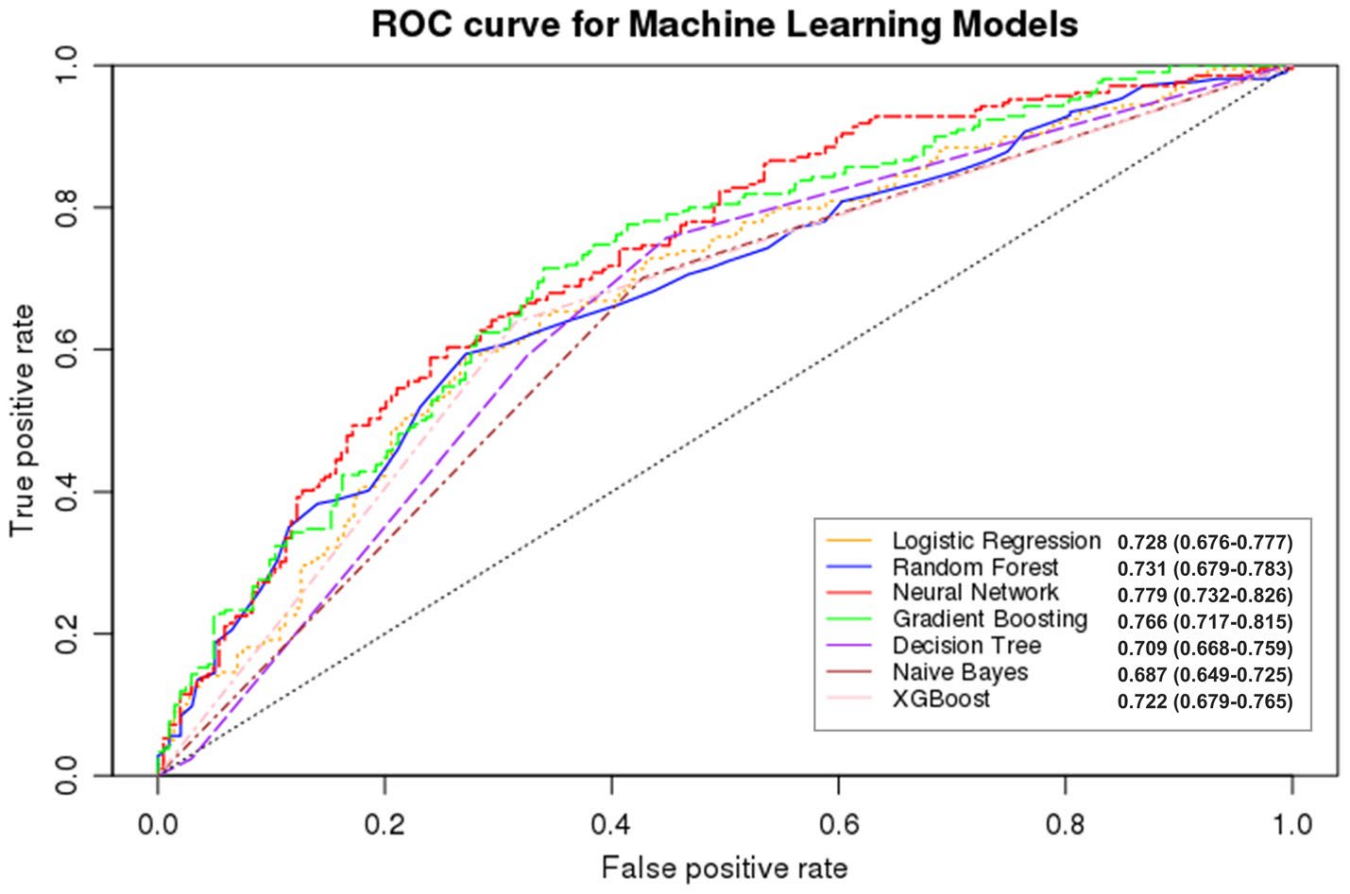
Receiver operating characteristic curve for the Parkinson’s disease prediction performance of each algorithm.

In model 3, the comparison of model performances showed that the model using a neural network algorithm exhibited the highest AUC (0.779; Fig. 1), followed by the gradient boosting machine (GBM) (AUC = 0.766) and random forest (AUC = 0.731). The accuracies of the neural network, GBM, and random forest algorithms were 0.687, 0.629, and 0.699, respectively. The performance of the PD prediction models for the algorithms is presented in Table 3.

**Table 3:** The performance of different Machine Learning Algorithms in Parkinson’s disease prediction (Model3).

Evaluation index	Logistic regression	Random forest	Neural network	GBM	Decision tree	Naïve Bayes	XGBoost
Accuracy	0.663	0.699	0.687	0.629	0.706	0.687	0.721
Sensitivity	0.728	0.683	0.677	0.45	0.807	0.751	0.698
Specificity	0.593	0.714	0.698	0.841	0.602	0.623	0.75
Precision	0.664	0.686	0.695	0.731	0.686	0.672	0.737
Recall	0.728	0.683	0.677	0.451	0.807	0.751	0.698
MCC	0.694	0.685	0.686	0.533	0.741	0.709	0.717
AUC	0.728	0.731	0.779	0.766	0.709	0.687	0.722

*GBM* Gradient Boosting Machines, *XGBoost* eXtreme Gradient Boosting, *MCC* Matthews correlation coefficient, *AUC* area under the receiver operating characteristic curve.

**Figure 2:**
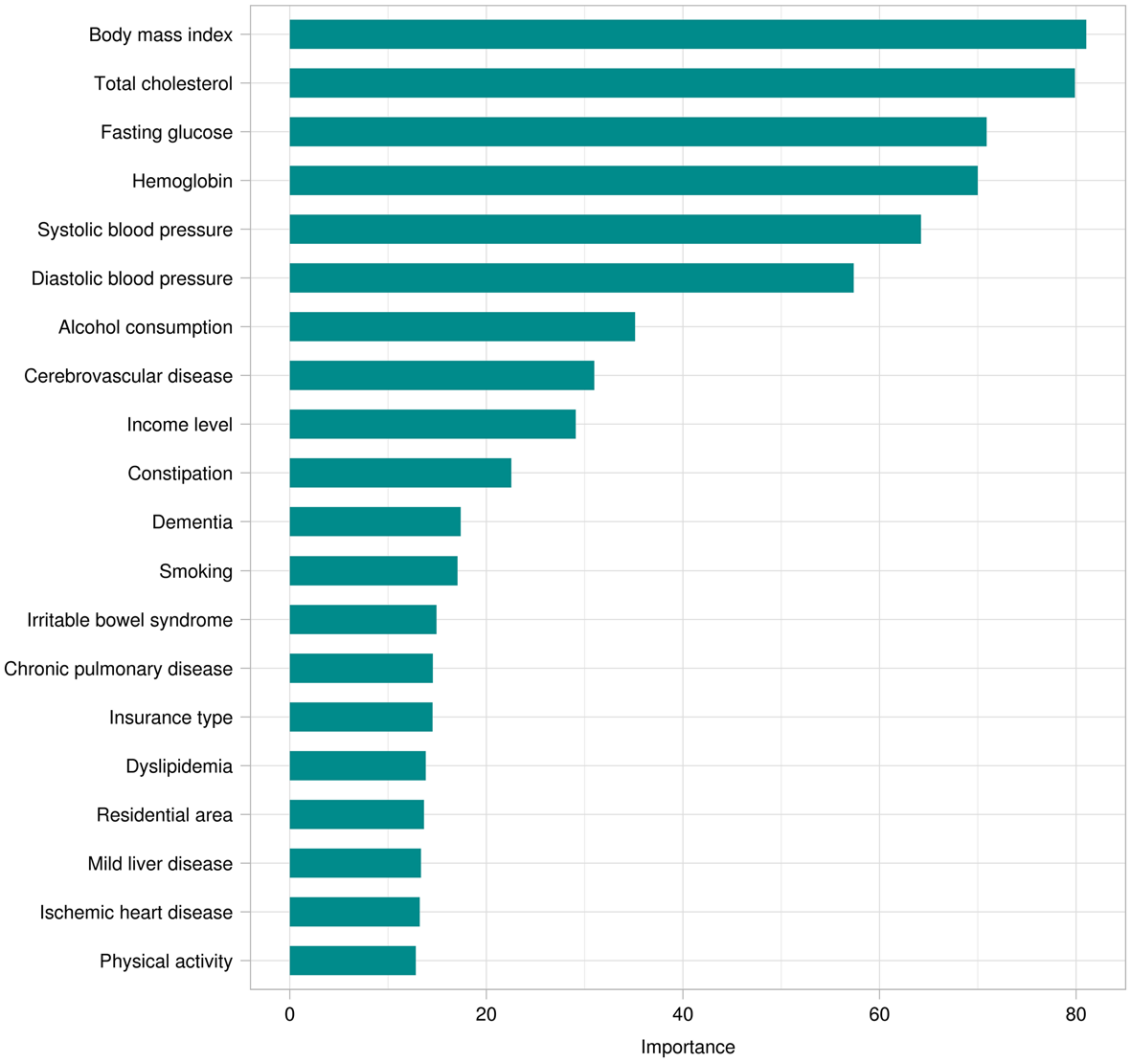
Feature importance for Parkinson’s disease prediction using a neural network algorithm.

We conducted variable selection using the random forest algorithm with permutation importance (Fig. 2). Of the 35 variables considered in this study, BMI was the most important contributing factor to PD prediction, followed by total cholesterol, fasting glucose, Hb, and blood pressure. Lifestyle factors (smoking, alcohol consumption, and PA) and SES (income level, insurance type, and residential area) were included in the top 20 factors. The top 20 comorbidities were cerebrovascular disease, constipation, dementia, irritable bowel syndrome, chronic pulmonary disease, dyslipidemia, mild liver disease, and ischemic heart disease.

### Subgroup analyses

**Figure 3:**
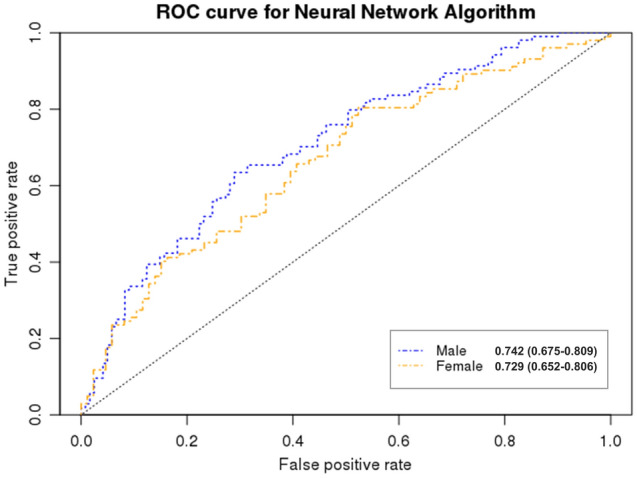
Receiver operating characteristic curve of the Parkinson’s disease prediction performance for the neural network algorithm according to sex.

**Figure 4:**
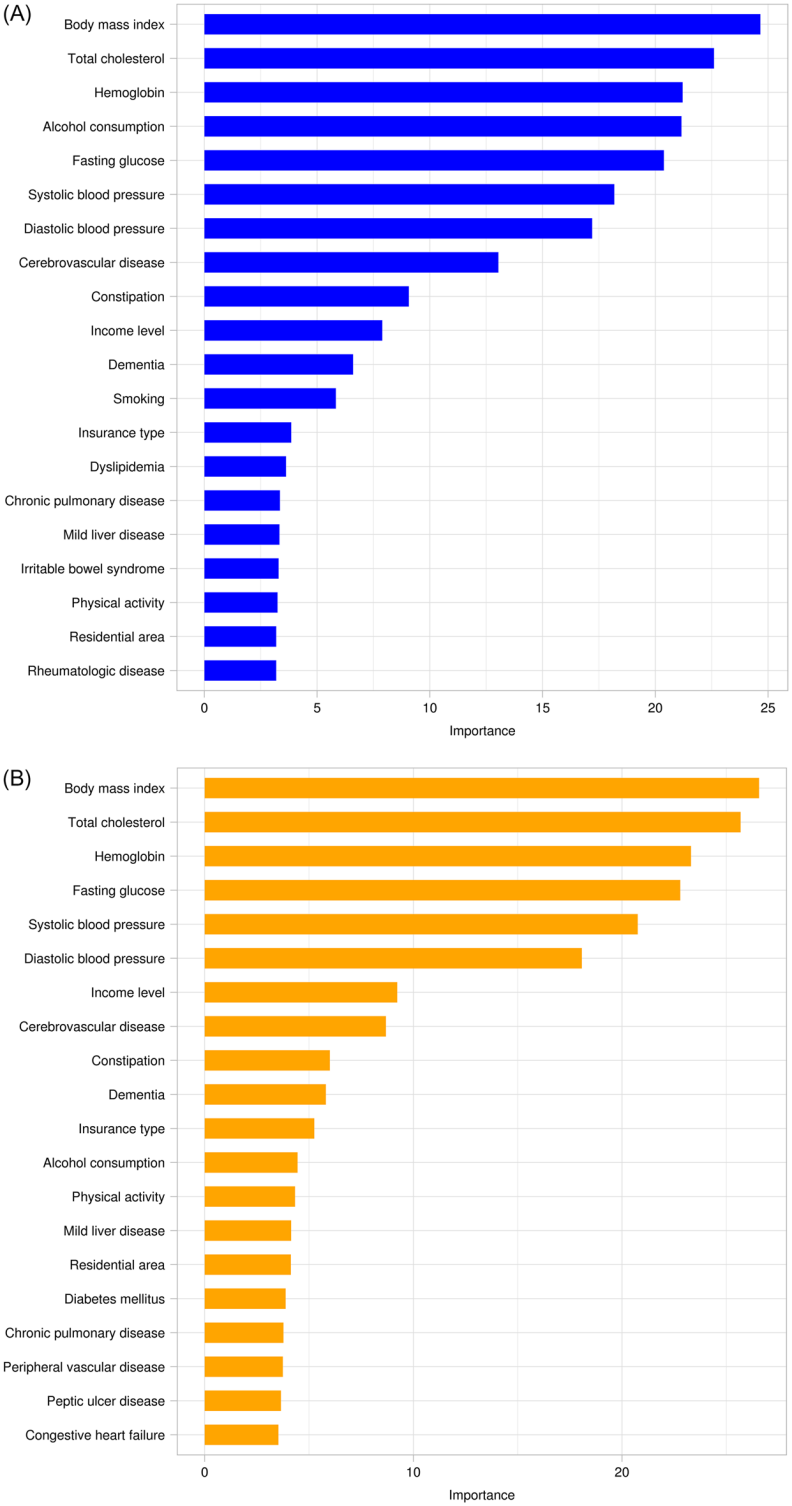
Feature importance for Parkinson’s disease prediction in the neural network algorithm by sex. (**a**) Male. (**b**) Female.

We used model 3 to perform a neural network algorithm analysis based on sex, which included SES, comorbidities, lifestyle factors, and anthropometric and laboratory data. The AUC and accuracy of the predictive model were 0.742 and 0.679 (for men) and 0.729 and 0.661 (for women), respectively. Overall, most model performance metrics were higher for men than for women. (Fig. 3, Table 4). Figure 4 displays the feature importance for the PD prediction model using the neural network algorithm according to sex. BMI was the most important predictive factor for PD development in both sexes, followed by cholesterol and Hb levels. There were some differences in feature importance between the sexes. Alcohol consumption and smoking were the top factors in men than in women, and smoking was not included in the top 20 predictive factors for women in the PD prediction model. In women, PA and DM were more highly ranked in terms of feature importance in the PD prediction model than in men. SES, including income level, insurance type, and residential area, was included as a predictive factor in both sexes, and each SES factor was more highly ranked in the PD prediction model in women than in men.

**Table 4:** Evaluation of Neural Network Algorithm in Parkinson’s disease prediction stratified by Sex.

	Accuracy	Sensitivity	Specificity	Precision	Recall	MCC	AUC
Male	0.679	0.727	0.638	0.635	0.727	0.678	0.742
Female	0.661	0.555	0.786	0.743	0.555	0.633	0.729

*MCC* Matthews correlation coefficient, *AUC* area under the receiver operating characteristic curve.

## Discussion

We analyzed the data of 2204 matched patients extracted from the NHIS-healS database: 1102 in the PD group and 1102 in the comparison group. We proposed a data-driven ML model that predicts PD occurrence using population-based, repeatedly measured health-screening data. The model performance was highest with the neural network algorithm, followed by GBM and random forest. The neural network, GBM, and random forest algorithms exhibited average AUCs of 0.779, 0.766, and 0.731, respectively. When analyzed separately by sex, for PD prediction ability, the AUC of the neural network algorithm was 0.742 and 0.729 in men and women, respectively, and the overall model performance metrics were higher in men than in women. The most important contributing factor for PD prediction was BMI, followed by total cholesterol, fasting glucose, Hb, and blood pressure levels. Smoking and alcohol consumption (in men) and SES, PA, and DM (in women) were highly correlated with PD.

The diagnosis of PD is commonly based on medical observations and the assessment of clinical signs, including the characterization of a variety of motor symptoms. Although non-motor symptoms of PD precede motor symptoms, various non-specific non-motor symptoms can be overlooked, making the diagnosis of PD challenging at an early stage. PD is one of the most common neurodegenerative diseases and has heterogeneous clinical outcomes. Hence, highly accurate predictive models are required for early detection and treatment guidance. In a previous study of an ML-based PD prediction model using demographic data and various comorbidities, the authors suggested that PD could be identified five years prior to PD diagnosis^10^. Several ML models exist for predicting PD using neuroimaging or video recordings; however, health record data-based prediction models for PD are limited. Herein, we used the NHIS-healS database, which covers over 500,000 representatives of the Korean population, to construct an ML model for PD prediction. In the database, various factors including demographic and lifestyle factors, SES, and comorbidities, which have rarely been considered when estimating PD risk, were included. Additionally, the NHIS-healS database contains longitudinal health-screening data, which allows the evaluation of the effects of time-varying covariables for PD prediction. Another strength of our study is that we suggested a cost-effective model for PD prediction. Using existing large-scale claims data for the ML model instead of collecting new data saved time and obviated additional costs and burdens.

This study shows that our ML algorithm for PD risk could be a useful option for PD prediction. Hall et al. investigated a PD prediction model using clinical and demographic data, family history, and genetic information and obtained an AUC of 0.73^13^. According to a study by Mei et al., the average AUC of the ML prediction model of a CSF-based study was 0.8^8^. The prediction performances of our ML algorithm for PD risk were similar to the results of previous prediction models that used various data such as demographic data, genetic information, laboratory data, and motion analysis^8,13,14^. Some previous studies have shown superior AUC values than our results; however, most of these studies required complex and expensive tests, including motion analysis using wearable multimodal sensors and diffuse tensor imaging^15^. Herein, we created three predictive models to determine how the performance of the ML algorithms changed according to various factors included in the analyses. Model performance generally increased as more variables were included in the ML algorithms. According to the neural network algorithm, the AUCs of models 2 and 3 were 0.767 and 0.779, respectively. Based on our results, even though the inclusion of anthropometric and laboratory data could increase prediction accuracy, PD could also be predicted with high accuracy using only clinical and lifestyle data that are more easily obtainable.

The most important contributing factor to predicting PD was BMI, followed by total cholesterol, fasting glucose levels, and Hb levels. The correction of the risk factors identified in this study needs more attention to predict the timing of PD onset accurately. In our study, BMI was the most important factor contributing to the occurrence of PD. Weight loss has been suggested to be a frequent non-motor symptom in the prodromal stage and during PD progression^16,17^. Individuals with PD began to lose weight 2–4 years before clinical diagnosis^18^. There was a significant difference in BMI values between the PD and control groups in our study, which is consistent with previous studies. Hence, our ML model showed that BMI was an important contributor to PD occurrence; therefore, weight loss in elderly people requires more attention, and appropriate nutritional support is warranted. The total cholesterol and Hb levels were lower in the PD group than in the control group. Many studies have inconsistently reported an association between PD and laboratory data, including cholesterol and Hb levels. High cholesterol level is related to a lower risk of PD^19^, whereas statins (used to reduce cholesterol levels) are a protective factor against PD^20^. Recently, a statin-free cohort study in Israel showed that higher total cholesterol levels indicated a reduced risk of PD^21^. A population-based cohort study in Taiwan showed that newly diagnosed anemia increased the risk of PD^22^. In contrast, another nationwide cohort study in Korea found that anemia was associated with a lower risk of PD, particularly in patients with moderate-to-severe anemia^23^. Herein, anemia was defined as an Hb level of < 13 g/dL for men and < 12 g/dL for women. Although the Hb level in the PD group in our study was slightly lower than that in the comparison group, the exact value was 13.43 g/dL, which cannot be defined as anemia. Thus, based on our ML model, low Hb levels could be associated with PD risk, and the severity of anemia, PD risk, and related pathophysiology need to be further investigated.

The third contributing factor for PD development was the fasting glucose level. DM is associated with the development of PD, motor progression, and cognitive decline after diagnosis^24^. Additionally, glycemic status has been suggested to be associated with PD risk, which is consistent with our findings^25^. According to our ML-based prediction model for PD, blood pressure was one of the important contributing factor. Previous studies on the association between hypertension and PD risk have reported inconsistent results^26^. Additionally, only a few studies have directly investigated the relationship between blood pressure and PD risk, which showed no significant association^27,28^. Hypertension and PD are prevalent in older adults, and more studies focusing on blood pressure and PD risk are needed.

Overall, the performance of the ML model for PD prediction was higher in men than in women. Factors affecting the occurrence of PD also differed according to sex. Previous studies have suggested that different mechanisms may be involved because the incidence and progression of PD differ according to sex^29,30^. There are clear sex-related differences in the epidemiological and clinical features of this disease. PD affects men twice as often as it affects women; however, women have a higher mortality rate and faster disease progression^31^. For PD prediction using the ML model, lifestyle factors, including smoking and alcohol consumption, were more strongly related to PD risk in men, and DM, PA (related to metabolic syndrome), and SES were more strongly related to PD risk in women. However, the association between SES, including income level, insurance type, residential area, and PD risk, has rarely been investigated. SES has a significant relationship with healthcare-seeking behaviors. Additionally, a previous study showed that the duration from symptom onset to movement disorder specialist visit was longer among women than in men^32^, which could partly explain the different associations between SES and PD diagnosis according to sex. Both genetic and environmental factors could affect the differences related to PD according to sex, and future studies on the pathophysiological mechanisms of sex-related differences in PD are warranted.

## Limitations

This study had several limitations. First, it was conducted in a population of the same race. Since the prevalence of PD differs according to race, future studies focusing on ML models for PD prediction in different races are warranted to generalize our results. Second, there may have been a selection bias. We only included individuals with PD who underwent more than five health screenings before PD diagnosis. Thus, it is possible that individuals with PD whose disability level was relatively mild or who had healthcare-seeking behaviors were enrolled in the analysis^33,34^. Third, a recall bias is possible. We collected data on smoking, alcohol consumption, and PA from self-reported questionnaires. Fourth, the operational definition of PD was based on the ICD-10 codes. This study used nationwide claims data; thus, clinical information, including motor symptoms or PD subtypes, was unobtainable. Instead, we only included individuals with a PD diagnosis of more than three times and excluded individuals with a combined diagnosis of secondary parkinsonism or atypical parkinsonism to increase diagnostic validity.

## Methods

### Data source

Korea has maintained a nationwide health insurance system since 1963 under the Korean NHIS, and nearly all data in the health system have been centralized in large databases. This data includes a unique anonymous number for each patient and summarizes age, sex, type of insurance, a list of diagnoses according to the International Classification of Diseases, Tenth Revision (ICD-10), medical costs claimed, and prescribed drugs. The NHIS provides a biannual national health-screening program (NHSP) without any cost to all beneficiaries aged ≥ 40 years. The NHSP includes a self-reported questionnaire on health behavior, medical history, anthropometric measurements, and laboratory tests for Hb, fasting glucose, and cholesterol levels. This study used the NHIS-healS database, with approximately 510,000 people randomly selected from among those aged ≥ 40 years in 2002 and 2003. This study was approved by the Institutional Review Board of the Korea University Guro Hospital, which waived the requirement for informed consent.

### Study population

From the data of 512,836 Koreans in the health check-up database, we selected data from individuals who had participated in the NHSPs more than five times between 2002 and 2015. To define a diagnosis of PD, we first selected patients with a primary or secondary diagnosis of ICD-10 code G20. In 2004, the Korean government started operating a registration program for rare intractable diseases including PD. Thus, individuals diagnosed with PD between 2004 and 2005 were excluded to ensure that the PD group included only individuals with new PD episodes. To ensure diagnostic validity, we included only those individuals who visited the clinics more than three times with a diagnosis of PD and excluded those with a combined diagnosis of secondary parkinsonism or atypical parkinsonism (ICD-10 code: G21–23). Individuals without a PD diagnosis who took part in the NHSP more than five times before the last healthcare visit date were included as a comparison cohort, and the PD group and the comparison group were subjected to 1:1 age matching. Finally, 2204 individual datasets (1102 in the PD and 1102 in the comparison groups) were used to train and validate the ML algorithms of the prediction model (Supplemental Material 1).

### Health-screening data

All participants in the NHSP were required to fill out self-report questionnaires, which included questions on smoking status (never, ex-smoker, and current smoker), alcohol consumption, and PA. Current smokers were defined as those who had smoked ≥ 100 cigarettes in their lifetime. Alcohol consumption was categorized based on weekly frequency of drinking (none, ≤ 3 times/week, or ≥ 4 times/week). PA was categorized based on the weekly frequency of exercise (≤ 4 or ≥ 5 times/week). Anthropometric data, including height, weight, and blood pressure (systolic and diastolic), were assessed. BMI was calculated as the weight divided by height squared (kg/m^2^) and categorized into five groups according to the Asia–Pacific BMI criteria established by the Western Pacific Region of the World Health Organization: < 18.5, 18.5–23.0, 23–25, 25–30, and ≥ 30 kg/m^2^. Venous samples were drawn after an overnight fast to determine fasting plasma glucose, total cholesterol, and Hb levels. Regarding the results from more than five NHSPs, the average for continuous variables and the mode for categorical variables were set as representative values.

### Other variables

Age was categorized into five groups: 40–49, 50–59, 60–69, 70–79, and ≥ 80 years. Residential areas were categorized into urban and rural. NHI premium was used as a proxy measure of income because it is proportional to monthly income, including earnings and capital gains. The income deciles of enrolled individuals were categorized into four groups (Q1, Q2, Q3, and Q4, indicating all medical aid enrollees + 0–20, 21–50, 51–80, and 81–100 percentile of NHI enrollees, respectively). Comorbidity was defined using the Charlson Comorbidity Index, the validity of which has been confirmed, and other diseases, which are prevalent or known to be related to PD development, including dyslipidemia, osteoporosis, ankylosing spondylitis, gout, irritable bowel syndrome, inflammatory bowel disease, and constipation, were extracted using their ICD-10 codes^21,28,35,36^.

### Statistical analyses

Baseline clinical characteristics of the PD and comparison groups were compared using the Student’s *t*-test for continuous variables and the chi-squared test for categorical variables. All statistical analyses were performed using SAS (version 9.4; SAS Institute Inc., Cary, NC, USA) with the statistical significance level set at P < 0.05.

We built three predictive models to elucidate which factors could increase the performance of ML algorithms, and variables included in the models were age, sex, SES, BMI, blood pressure, fasting glucose, total cholesterol, Hb, smoking, alcohol consumption, PA, and comorbidities. Seven ML algorithms were implemented: logistic regression, random forest, neural network, GBM, decision tree, naïve Bayes, and eXtremeXGBoost. The training set was randomly partitioned into five subsets of almost equal size for five-fold cross validation. One partition was selected as the validation set, and the remaining partitions were used to train the predictive models. For feature selection, permutation importance was calculated by performing a random forest analysis, which is more appropriate for nonlinear classifiers^37^. We performed hyperparameter tuning, a process that adjusts an algorithm to improve the accuracy of the prediction model. Model performance metrics were measured with the test dataset using the AUC, accuracy, sensitivity, specificity, recall, and MCC^38,39^. The ML algorithms used in our study were developed using R version 3.3.3, including the packages caret^40^, neural network^41^, random forest^42^, e1071^43^, rpart^44^, GBM^45^, XGBoost^46^, ROCR^47^, and pROC^48^.

## Supplementary Information

Supplementary Figure 1.

Supplementary Legends.

## Data Availability

The corresponding authors take responsibility for the integrity of the data and the accuracy of the data analysis. The datasets generated during and/or analyzed during the current study are available from the corresponding authors on reasonable request.
