# Supplementary figures and images for: Machine learning based risk prediction for Parkinson's disease with nationwide health screening data
Source: Sci Rep. 2022 Nov 14;12:19499. doi: 10.1038/s41598-022-24105-9 (PMC9663430; doi:10.1038/s41598-022-24105-9)

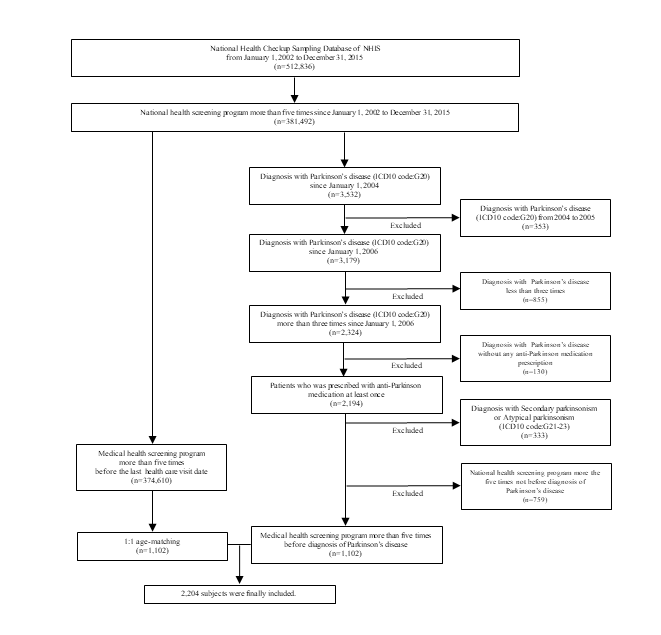

Supplement: Supplementary file 1 — Supplementary Figure 1. [file 41598_2022_24105_MOESM1_ESM.tif]
