# Supplementary material for: Machine learning based risk prediction for Parkinson's disease with nationwide health screening data
Source: Sci Rep. 2022 Nov 14;12:19499. doi: 10.1038/s41598-022-24105-9 (PMC9663430; doi:10.1038/s41598-022-24105-9)
Supplement: Supplementary file 2 — Supplementary Legends. [file 41598_2022_24105_MOESM2_ESM.docx]

**Supplemental Material**

Supplemental Material 1. Flowchart for sample selection.
